# PARADISE 24: A Measure to Assess the Impact of Brain Disorders on People’s Lives

**DOI:** 10.1371/journal.pone.0132410

**Published:** 2015-07-06

**Authors:** Alarcos Cieza, Carla Sabariego, Marta Anczewska, Carolina Ballert, Jerome Bickenbach, Maria Cabello, Ambra Giovannetti, Teemu Kaskela, Blanca Mellor, Tuuli Pitkänen, Rui Quintas, Alberto Raggi, Piotr Świtaj, Somnath Chatterji, PARADISE Consortium

**Affiliations:** 1 Faculty of Social and Human Sciences, School of Psychology, University of Southampton, Southampton, United Kingdom; 2 Department of Medical Informatics, Biometry and Epidemiology (IBE), Chair for Public Health and Health Services Research, Research Unit for Biopsychosocial Health, Ludwig-Maximilians-University (LMU), Munich, Germany; 3 Swiss Paraplegic Research, Nottwil, Switzerland; 4 Department of Psychiatry, Institute of Psychiatry and Neurology, Warsaw, Poland; 5 Instituto de Salud Carlos III, Centro de Investigación Biomédica en Red de Salud Mental (CIBERSAM), Department of Psychiatry, Universidad Autónoma de Madrid, Psychiatry Service, Instituto de Investigación del Hospital Universitario de La Princesa (IIS-IP), Madrid, Spain; 6 Department of Psychiatry, Universidad Autónoma de Madrid, Madrid, Spain; 7 Neurology, Public Health and Disability Unit, Scientific Directorate, Neurological Institute Carlo Besta IRCCS Foundation, Milan, Italy; 8 A-Clinic Foundation, Helsinki, Finland; 9 Multi-Country Studies, Department of Measurement and Health Information Systems, World Health Organization, Geneva, Switzerland; Albert Einsten College of Medicine, UNITED STATES

## Abstract

**Objective:**

To construct a metric of the impact of brain disorders on people’s lives, based on the psychosocial difficulties (PSDs) that are experienced in common across brain disorders.

**Study Design:**

Psychometric study using data from a cross-sectional study with a convenience sample of 722 persons with 9 different brain disorders interviewed in four European countries: Italy, Poland, Spain and Finland. Questions addressing 64 PSDs were first reduced based on statistical considerations, patient’s perspective and clinical expertise. Rasch analyses for polytomous data were also applied.

**Setting:**

In and outpatient settings.

**Results:**

A valid and reliable metric with 24 items was created. The infit of all questions ranged between 0.7 and 1.3. There were no disordered thresholds. The targeting between item thresholds and persons’ abilities was good and the person-separation index was 0.92. Persons’ abilities were linearly transformed into a more intuitive scale ranging from zero (no PSDs) to 100 (extreme PSDs).

**Conclusion:**

The metric, called PARADISE 24, is based on the hypothesis of horizontal epidemiology, which affirms that people with brain disorders commonly experience PSDs. This metric is a useful tool to carry out cardinal comparisons over time of the magnitude of the psychosocial impact of brain disorders and between persons and groups in clinical practice and research.

## Introduction

The prevalence of mental disorders such as depression, schizophrenia, and substance dependency, and neurological disorders such as dementia, headache and epilepsy—together called brain disorders—is extremely high. In Europe, for example, a multi-method study has estimated that over 38% of the total EU population (or more than 160 million people) suffer from at least one of the 27 brain disorders reviewed [1]. The burden of these disorders is also high, higher even than that of cardiovascular diseases and cancer [2,3]. Given that depression and dementia are age-related conditions and the highest contributors to the overall burden of brain disorders, the effect of demographic ageing trends in Europe and other parts of the world will dramatically increase this burden in the near future. Accordingly, brain disorders have been put to the forefront of the political and scientific agendas [1,2,4].

Disability-adjusted Life Years (DALYs), the measure used to calculate the burden of disease in the Global Burden of Disease studies, is a composite of years lost due to premature mortality and years lived with disability, understood as non-fatal health consequences of diseases. Although mortality in some brain disorders is moderately high, disability largely accounts for the burden of these conditions [3,5]. Yet, like all indirect measures, such as “health gap” or “health expectancy”, DALYs do not use data collected directly from people with health conditions, but rather relies on available population-based mortality and morbidity statistics and are calculated using disability weights. These weights are derived from evaluations of the extent of disability that the general public and health professionals attribute to summary descriptions of the health consequences of each disease at different severity levels [6]. DALYs, therefore, are only meaningful for comparisons at the population level, where they are important tools for health-policy development and resource allocation [7].

To understand the true impact of brain disorders on a person’s life, however, it is essential to collect information directly from people with such disorders. Direct information on this impact gives clinicians insight into the outcomes of treatment and provides the information they need to monitor disease processes and treatment management over time. For clinical and epidemiological researchers, this information is also indispensable to follow population trends and make cost-effectiveness evaluations, since treatments are only effective if they actually make a difference to the day-to-day lives of people.

There is, however, no direct measure or metric that captures the impact of brain disorders on people’s lives and based on which comparisons across brain disorders are possible. As described in Cieza et al. [8], what has been done so far is to operationalize this impact in terms of psychosocial difficulties (PSDs), such as sleep and memory problems and difficulties in maintaining relationships. In addition, support has been shown for the hypothesis of ‘horizontal epidemiology’, namely that a common set of PSDs are experienced across brain disorders. For that study, PSDs were defined using the framework of the International Classification of Functioning, Disability and Health (ICF) [9]. PSDs are impairments in mental functions and impairments in body functions under nervous-system control, activity limitations and participation restrictions that result from the interaction of a person with a brain disorder and the environmental and personal factors.

The question remains whether a direct metric of the impact of brain disorders on people lives that is operationalized in terms of PSDs can be constructed. A true metric can only be constructed psychometrically with Item-Response-Theory (IRT) approaches, which enable information about specific PSDs to be integrated into a single summary score. Clinicians and researchers can use these scores to monitor change over time and to evaluate the effectiveness of interventions. If the metric were constructed from PSDs experienced in common across brain disorders, as we propose in this investigation, it would be possible to compare the impact of different disorders and the outcomes of interventions across disorders.

The objective of our paper is thus to construct a true metric of the impact of brain disorders on people’s lives, based on the PSDs that are experienced in common across brain disorders, the existence of which confirmed the hypothesis of horizontal epidemiology.

## Methods

### Ethic statement

The study was conducted in conformity with the ethical principles of the EC Research Ethics Committee and approved by the Ethics Committee of the Ludwig-Maximilian University, Munich, Germany, which was the coordinating center, as well as by the Ethics Committee of the Neurological Institute Carlo Besta IRCCS Foundation in Milan, Italy, the Institute of Psychiatry and Neurology in Warsaw, Poland, the teaching hospital La Princesa of the University of Madrid in Madrid, Spain and the Järvenpää Addiction Hospital in Haarajoki, Finland.

### Design and sample

This is a psychometric study using data from a cross-sectional study carried out with a convenience sample of 722 persons with dementia (N = 80), stroke (N = 80), multiple sclerosis (MS) (N = 80), epilepsy (N = 80), migraine (N = 80), Parkinson′s Disease (PD) (N = 80), depression (N = 81), schizophrenia (N = 81) or substance dependency (N = 80). 289 were in and 392 outpatients. Twenty four had other living situation. The data was collected in the scope of the EU-funded project “Psychosocial fActors Relevant to BrAin DISorders in Europe” (www.paradiseproject.eu) [8]. Patients were interviewed by a trained clinical researcher using the PARADISE data collection protocol developed in the project. The original protocol included 64 PSDs and 59 PSDs determinants considered to be common across brain disorders as well as questions targeting demographic information, age, the impact of comorbidities, and standard disorder-specific measures routinely used to assess disease severity. Persons with stroke, MS, epilepsy, migraine and PD were recruited at the Neurological Institute Carlo Besta IRCCS Foundation in Milan, Italy; persons with dementia and schizophrenia at the Institute of Psychiatry and Neurology in Warsaw, Poland; persons with depression at the teaching hospital La Princesa in Madrid, Spain; and persons with substance-dependency at the Järvenpää Addiction Hospital in Haarajoki, Finland.

Individuals participating in the study had to meet the following general inclusion criteria: age ≥ 18 years; main diagnosis (according to ICD-10) of one of the disorders listed above; and the individual had been informed of the purpose and rationale of the study and had signed the “patient consent form”. The sample is described in detail elsewhere [8].

The original version of the PARADISE data-collection protocol included 64 PSDs and 59 determinants of those PSDs, but only data referring to the 64 PSDs were considered for this study. All these PSDs had been operationalized with questions from questionnaires, clinical instruments and national and international health surveys. If no standard question was available, new questions were developed. The response options were homogenized to be the same for all PSDs questions, namely “None”, “Mild”, “Moderate”, “Severe”, “Extreme”, “Don’t know” and “Not applicable”. Don’t know” was included to record the percentage of persons not able to choose a response option, i.e. was considered a measure of how understandable questions are; “Not applicable” was included to evaluate how universal questions are to our sample with brain disorders, i.e. to which percentage of respondents they do not apply. The protocol also included a section in which patients were asked to mention the up to five PSDs that were most salient to them.

### Data preparation

For this psychometric investigation, the response options “Don’t know” and “Not applicable” were considered missing values. The percentage of missing values was extremely low (<3.5%) for all but six PSD. Three PSDs (Libido, Independence in everyday activities, and Caring for others) with high percentages of “not applicable” or “don’t know” had missing rates between 7.0 and 8.5%, and three PSDs (Driving, Sexual functions, and Education / Work and employment) had a very high percentage of “not applicable” responses, resulting in missing-value rates between 21.0 and 41.4%. We did not consider this to be a problem for the analyses because the estimations carried out with the Rasch model readily deal with missing values [10].

### Data analysis

The metric of the impact of brain disorders on people’s lives was developed in two phases.

The objective of the ***first phase*** was to reduce the 64 PSDs of the PARADISE data-collection protocol to a number that makes the metric more feasible for clinical practice and research. The number of PSDs was reduced by clinicians and researchers working in the field of brain disorders during a two-day workshop based on the following four criteria:

1. commonly experienced across brain disorders as described in Cieza et al. [8],
2. non-redundant, i.e. do not correlate highly with other PSDs,
3. representing the whole continuum of PSDs according to Rasch analyses [11], and
4. free of Differential Item Functioning (DIF).

Especially in those cases in which a clear selection based on these criteria was not possible, two additional criteria were taken into account: a) those PSDs which were mentioned by the patients as the most salient during the interviews were prioritized, and b) the clinical expertise of the project team was taken into account, and those PSDs considered most relevant for persons with brain disorders from a clinical point of view were also prioritized.

To be able to apply criterion 2, polychoric correlations were estimated [12,13]. This type of correlation assumes ordinal-response options to be a categorized representation of an underlying continuous variable and estimates the correlation of those underlying continuous variables. Correlation coefficients of r>0.9 were considered high and an indication of redundancy.

To be able to apply criteria 3 and 4, Rasch analyses for polytomous data (also known as Partial Credit Model) [11,14] were carried out with the 64 PSDs of the PARADISE data-collection protocol. The Polytomous Rasch Model is an IRT Model based on the assumption that there is a unidimensional latent construct to be measured and that both persons and items can be located along the continuous unidimensional latent construct. Information about the latent construct is obtained from the persons’ responses to items, e.g. questions with ordinal-response options from a questionnaire [15]. The model’s calculations lead to information refereeing of both persons and items. Each person’s so-called ability is obtained, i.e. the location of the person on the continuum. For each item, the so-called item difficulty is obtained, i.e. the location of the item on the continuum. In addition, item thresholds are available for each item. For an item with k response options, there are k-1 thresholds. These indicate the location of the latent trait where the response options of items best discriminate between persons.

Item thresholds and persons’ abilities should reveal good **targeting**, i.e. the estimated item thresholds should cover the same range on the continuum as the estimated persons’ abilities and be evenly distributed in that range. Items with very similar thresholds are considered redundant, so that one can be deleted without losing information necessary to estimate the persons’ abilities.

Within the framework of the Rasch model, items should work in the same way, irrespective of the group being assessed, i.e. the difficulty of an item should be the same regardless of e.g., gender. Items that violate this criterion exhibit Differential Item Functioning (DIF).

We carried out the Rasch analyses and paid special attention to the results of item difficulty, i.e. item thresholds (criterion 3), and DIF (criterion 4). Bi-factor analysis was used to verify unidimentionality. In bi-factor analysis an extra factor, i.e., a general factor that loads in all items is estimated and unidimensionality was considered to be met, if all questions used in the instrument load higher in the general factor than in the specific factors. Prior to bi-factor analyses we estimated, in a first step, polychoric correlation coefficients for ordered-category data. In a second step, Parallel Analysis was carried out in order to decide how many factors should be retained in the Bi-Factor Analysis. In parallel analysis the eigenvalues resulting from an exploratory factor analysis and based on the polychoric correlations of the actual data are compared to those resulting from simulated data. The number of factors is defined as the number of eigenvalues from the actual data exceeding those of the simulated data. Bi-factor analysis was then carried out with this number of factors.

We used the lordif package in R to test for DIF for gender and psychiatric vs. neurologic disorders. This package performs iterative hybrid ordinal logistic regression and uses the persons’ ability parameters as conditioning variable; change in McFadden’s pseudo R-squared measure (>0.02) was used as the DIF criterion [16,17]. Items not showing DIF were preferred over items showing DIF wherever possible.

The objective of the ***second phase*** was to create a metric of the impact of brain disorders on people’s lives and to evaluate its psychometric properties based on the selected subset of PSDs. We again applied Rasch analysis and examined the following properties: item fit, ordering of the thresholds, targeting between item thresholds and persons’ abilities, DIF and reliability.

**Item fit** was examined based on the infit mean square statistics. The infit should fall between 0.7 and 1.3 to indicate good item fit [18]. The **ordering of item thresholds** was studied based on the threshold estimates for each PSD. The items’ thresholds should have increasing values. If this was not the case and items’ thresholds were disordered, response options for those items have to be collapsed as recommended by Andrich 2005 and Linacre 2002 [19,20]. The **targeting** between item thresholds and persons’ abilities was examined by comparing the distribution of persons’ abilities and item thresholds along the latent trait continuum. If both are in the same range of the continuum, the set of items is well targeted. **DIF** was tested again for gender and psychiatric vs. neurologic disorders using the same methodology as described above. **Reliability** was studied with the Person Separation Index r_ß_, which is analogous to the traditional test theory indices Kuder-Richardson Formula 21 or Cronbach’s alpha and ranges between zero and 1, where the value of 1 indicates perfect reproducibility of person placements [21].

Finally, persons’ abilities—originally obtained on a logit scale—were linearly transformed to a more intuitive scale ranging from zero (no PSDs) to 100 (extreme PSDs) [22].

Data analyses were performed in SPSS, SAS and R.

## Results

Table 1 summarizes the demographic characteristics and disease severity of the sample.

**Table 1:** Demographic characteristics of the sample.

		Epilepsy	Migraine	Multiple Sclerosis	Parkinson	Stroke	Dementia	Depression	Schizophrenia	Substance Dependency
**N**		80	80	80	80	80	80	81	81	80
**Age (years)**	**Mean**	41,23	44,54	41,03	61,24	59,84	81,03	54,81	38,38	39,56
	**SD**	11,99	12,12	8,74	10,45	14,36	5,49	14,73	14,03	13,15
**Gender (%)**	**Female**	50,0%	86,3%	65,0%	40,0%	43,8%	78,8%	82,7%	53,1%	37,5%
**General living situation (%)**	**Living independently and alone**	11,3%	12,5%	15,0%	13,8%	10,0%	25,0%	34,6%	23,5%	41,3%
	**Living independently with others in a household**	88,8%	87,5%	83,8%	86,3%	83,8%	55,0%	59,3%	65,4%	42,5%
**Persons working (%)**		66,3%	67,5%	72,5%	33,8%	25,0%	0,0%	27,2%	8,6%	6,3%
**Disease duration (years)**	**Mean**	18,67	21,13	7,66	6,26	4,00	3,69	12,63	13,03	12,16
	**SD**	12,32	14,60	6,94	4,40	6,48	2,70	11,57	11,83	8,67
**Disease severity**	**Instrument** *	**CRS**	**MIDAS**	**EDSS**	**Hoehn & Yahr**	**NIHSS**	**MMSE**	**HDRS**	**CGI**	**ADS** **
	**N**	79	80	80	80	55	80	81	81	34
	**Mean (SD)**	na	27,16 (22,92)	2,13 (1,74)	na	4,93 (4,39)	21,10 (2,89)	19,70 (5,49)	na	
	**Cut-off Mild Severity**	**= 1**	**<6**	**<3**	**= 1 or 1.5**	**1 to 5**	**≥25**	**<14**	**= 2 or 3**	**≤ 13**
	**No of persons**	24	9	64	15	37	6	11	32	1
	**Cut-off Moderate Severity**	**= 2**	**≥ 6 & ≤ 20**	**≥ 3 & ≤ 5**	**= 2 or 2.5**	**6 to 14**	**≥ 10 & < 25**	**≥ 14 & ≤ 18**	**= 4 or 5**	**≥ 14 & ≤ 21**
	**No of persons**	28	27	9	58	14	74	26	49	7
	**Cut-off High Severity**	**= 3**	**>20**	**> 5**	**= 3 or 4**	**≥ 15**	**< 10**	**>18**	**= 6 or 7**	**≥ 22**
	**No of persons**	27	44	7	7	29	0	44	0	26

* HDRS: Hamilton Depression Rating Scale; CRS: Clinical Rating of Severity; MIDAS: Migraine Disability Assessment; EDSS: Expanded Disability Status Scale; Hoehn & Yahr: Hoehn & Yahr Score; NIHSS: National Institutes of Health Stroke Scale; CGI: Clinical Global Impression (CGI); MMSE: Mini Mental State Examination; ADS: Alcohol Dependence Scale.

** In substance dependency, 44 persons had alcohol dependence as their main diagnosis. The data reported here refer to the 34 of those from whom the ADS data were available. Mean is not reported because of the low N. For all other substance dependency conditions, the intention was to collect data with the ‘Severity of Dependence Scale’. There were, however, a larger number missing data and the results are, therefore, not reported.

Bi-Factor analysis was carried out in order to verify the assumption of unidimensionality, a requirement to apply Rasch Analyses. Estimated polychoric correlation coefficients confirmed the absence of highly correlated variables (r>0.95) and parallel analysis indicated that the number of factors in the bi-factor analysis should equal 10. Bi-Factor analysis was therefore carried out with 10 factors. Factor loadings of the bi-factor analysis with 10 specific factors and an extra general factor showed that the factor loading on the general factor was consistently higher than the loading on the specific factors. The assumption of unidimensionality was considered to be met and Rasch Analysis carried out will all 64 items.

In the ***first phase***, during the two-day workshop and after applying the four above-mentioned criteria, the number of PSDs to be considered in the metric of the impact of brain disorders on people’s lives was reduced from 64 to 24. The 64 PSDs are reported in Cieza et al. and a table including the PSD of the PARADISE data collection protocol and the percentage of persons reporting PSDs by health disorder is available as supplementary file (S1 Table). [8]. The 24 PSDs selected together with the category of the ICF that they represent, as well as the question used to operationalize them, are presented in Table 2.

**Table 2:** Item characteristics resulting from the final Rasch model: infit statistics, item location, and item thresholds.

****ICF Code****	****PSD Name****	****Question****	****Infit****	****Location****	****Threshold 1****	****Threshold 2****
**Mental functions**						
b130	Energy and drive functions	How much of a problem did you have due to not feeling rested and refreshed during the day (e.g. feeling tired, not having energy)?	0.849	-0.829	-2.545	0.888
b1301	Motivation	How much of a problem did you have not finding things that kept you interested and motivated?	0.814	-0.067	-0.966	0.832
b1302	Appetite	How much of a problem did you have with your appetite?	1.067	0.848	0.379	1.317
b134	Sleep functions	How much of a problem did you have with sleeping, such as falling asleep, waking up frequently during the night or waking up too early in the morning?	1.152	-0.227	-1.201	0.747
b140	Attention functions	How much difficulty did you have in concentrating on doing something for ten minutes?	0.871	0.509	-0.523	1.542
b144	Memory functions	How much difficulty did you have in remembering to do important things?	1.062	0.115	-1.056	1.286
b147	Psychomotor functions	How much of a problem did you have with being slowed down or feeling as if things were moving too fast around you?	0.984	0.036	-1.232	1.303
b147	Agitation & Aggression / Hyperactivity	How much of a problem did you have being so irritable that you started arguments, shouted at people or even hit people?	1.124	0.571	-0.250	1.391
b152	Depressive mood	How much of a problem did you have with feeling sad, low or depressed?	0.796	-0.630	-1.886	0.627
b152	Worry and anxiety	How much of a problem did you have with worry or anxiety?	0.912	-0.721	-1.934	0.492
b152	Stress	How much of a problem did you have with not being able to cope with all the things that you had to do?	0.836	-0.228	-1.332	0.876
b164	Executive functions	How much difficulty did you have in making decisions?	0.834	0.314	-0.569	1.197
**Other body functions under central neurological control**						
b280	Pain	How much bodily ache or pain did you have?	1.188	-0.120	-1.229	0.989
b640	Sexual functions	How much difficulty did you have in sexual activities?	1.131	0.494	0.472	0.515
**Difficulties in activities and participation**						
d3	Communication	How much difficulty did you have in starting and maintaining a conversation?	1.001	0.912	-0.017	1.841
d450	Walking	How much difficulty did you have in walking a long distance such as a kilometre (or equivalent)?	1.257	0.395	-0.175	0.964
d510 + d530 +d540 + d550	Self-care	How much difficulty did you have in grooming or dressing, toileting or eating?	0.986	0.936	0.287	1.586
d5	Independency in everyday activities	How much difficulty did you have in staying by yourself for a few days?	1.075	0.688	0.629	0.747
d570	Looking after one’s health	How much difficulty did you have with looking after your health, such as eating well, exercising and taking your medicines?	0.906	0.791	0.270	1.311
d7500	Informal relationships with friends	How much difficulty did you have in maintaining a friendship?	0.921	0.890	0.243	1.536
d760 + d770	Family relationships and intimate relationships	How much difficulty did you have in getting along with people who are close to you?	0.935	1.195	0.192	2.199
d839 + d850	Education / Work and employment	How much difficulty did you have in your day-to-day work or school?	0.992	-0.202	-1.113	0.708
d870	Economic self-sufficiency	How much difficulty did you have with managing your money?	1.040	0.859	0.247	1.471
d9	Community, social and civic life	How much difficulty did you have in joining in community activities (for example, festivities, religious or other activities) in the same way as anyone else can?	0.808	0.220	-0.186	0.625

Even though the PSDs addressing specific areas of self-care (Washing oneself, Toileting, Dressing and Eating) were not frequently experienced across brain disorders (criterion 1) when considered separately, the participants in the workshop decided that a PSD on general self-care should be selected. Data on this PSD was generated combining the data of all 4 specific self-care PSDs and using the highest level of limitation each person reported in all 4. The newly created PSD called general self-care was experienced across brain disorders according to the criteria of Cieza et al. [8]. The participants also agreed that the question to be used in future studies to operationalize general self-care should be “*How much difficulty did you have in grooming or dressing*, *toileting or eating*?”

The participants also made the following recommendations for the questions used to operationalize the following PSDs based on the data-collection experience:

1. Looking after one’s health: the word ‘*prescribed*’ should be added to medicines *(How much difficulty did you have with looking after your health*, *such as eating well*, *exercising and taking your prescribed medicines*?)
2. Informal relationships with friends: ‘*maintaining a friendship*’ should be replaced by ‘*initiating and maintaining a friendship*’ (*How much difficulty did you have in initiating and maintaining a friendship*?)

In the **second phase**, an initial Rasch model with the questions operationalizing the 24 PSDs selected in the first phase was calculated. Twelve questions presented disordered thresholds, and one item ‘*How much difficulty did you have in walking a long distance*, *such as a kilometre (or equivalent)*?’ presented DIF for neurological/psychiatric conditions.

With respect to the disordered thresholds, we decided to reduce the number of response options from 5 to 3 using the collapsing strategy 01122 for all questions (Mild collapsed with Moderate and Severe with Extreme). This decision was not only made based on a) the large number of questions with disordered thresholds, b) the frequencies of the response options and c) which response options presented disordered thresholds, but also on the fact that any measure is much more feasible and easier to fill in when all questions have the same response options.

With respect to the DIF of the question on walking, we had two options: delete the item, or split the item calculating its difficulty separately for neurological and psychiatric conditions. Deleting was not an option because this question was the only one directly addressing difficulties in mobility. Splitting the item was not ideal because it would force us to create two separate converting tables from the logic scale to a scale from 0 to 100 for neurological and psychiatric conditions. This would reduce the feasibility and the simplicity of the measure. We therefore decided to keep the question in the measure as it was for the sake of feasibility and simplicity, accepting the arguably small measurement error resulting from this decision.

The Rasch model was calibrated again after collapsing the response options. Table 2 presents the items’ locations, infit estimates and their thresholds. The infit of all questions ranges between 0.7 and 1.3, which indicates good item fit. There are no disordered thresholds.

The question on walking still presented DIF for neurological/psychiatric conditions. For the same reason as before, no further actions were taken to resolve this problem.

**Fig 1:**
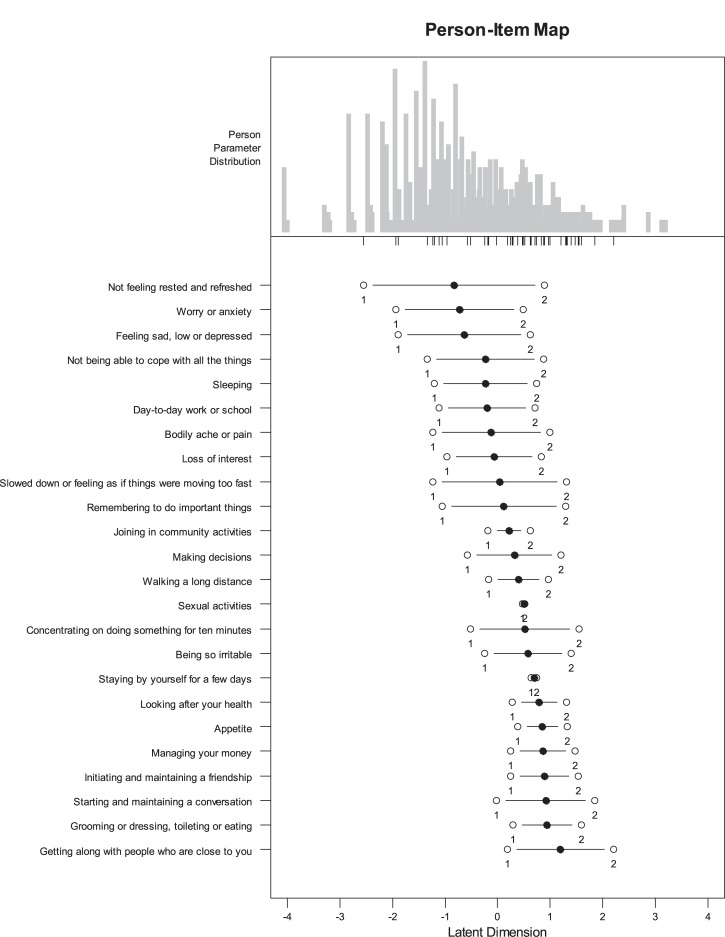
Distribution of persons’ abilities, items’ difficulties (bullets) and items’ thresholds (circles) on the latent scale. Legend: The upper part of the figure displays the distribution of personal abilities, while items’ locations and thresholds are displayed on the lines below. The items are presented according to their location in increasing order.

The targeting between item thresholds and persons’ abilities is shown in Fig 1. The targeting is good. The thresholds cover the whole continuum of PSDs with more threshold density in the higher levels of the continuum (towards a higher degree of difficulties). The person-separation index was 0.92, which indicates a high reliability and reproducibility of persons’ placements with the developed metric.

Persons’ abilities were linearly transformed into a more intuitive scale ranging from zero (no PSDs) to 100 (extreme PSDs). Table 3 presents the assignment of raw scores to persons’ abilities on the logic scale and to metric scores ranging from 0 to 100. This table is an aid for all users of the developed measure to calculate an intuitive metric score from 0 to 100 of the impact of brain disorders on people’s lives with which true comparisons over time between individuals or groups can be made. For everyday use of the metric, the second column regarding persons’ abilities can be omitted.

**Table 3:** Assignment of raw scores, persons’ abilities and transformed scores on a 0–100 scale of the PARADISE 24 metric.

****Raw scores****	****Persons' abilities****	****Transformed score****
0	-4.898	0
1	-4.065	10
2	-3.303	19
3	-2.832	25
4	-2.481	29
5	-2.196	33
6	-1.955	36
7	-1.743	38
8	-1.554	41
9	-1.382	43
10	-1.223	45
11	-1.076	46
12	-0.938	48
13	-0.807	50
14	-0.683	51
15	-0.565	53
16	-0.452	54
17	-0.343	55
18	-0.237	57
19	-0.135	58
20	-0.035	59
21	0.063	60
22	0.159	61
23	0.254	63
24	0.348	64
25	0.441	65
26	0.534	66
27	0.627	67
28	0.720	68
29	0.815	69
30	0.910	71
31	1.007	72
32	1.107	73
33	1.209	74
34	1.315	76
35	1.424	77
36	1.539	78
37	1.660	80
38	1.793	81
39	1.937	83
40	2.087	85
41	2.242	87
42	2.397	89
43	2.552	91
44	2.707	92
45	2.862	94
46	3.016	96
47	3.171	98
48	3.326	100

The final measure presented in the supplementary file (S1 Text) can be used in clinical practice and research and integrates the rewording of three items proposed by the persons involved in its development and the newly-created item on self-care.

## Discussion

We constructed a valid and reliable metric with which information directly collected from persons with brain disorders can be integrated in a single score to estimate the impact of those disorders on people’s lives. We call this metric PARADISE 24 because it has been developed within the scope of the EU-funded project “Psychosocial fActors Relevant to BrAin DISorders in Europe”, that uses the acronym PARADISE. To our knowledge, this is the first such metric constructed to make comparisons over time and between persons and groups of persons with different brain disorders.

Our approach is original because it was guided by the hypothesis of horizontal epidemiology, namely that there are PSDs experienced in common across brain disorders [8]. The selection of PSDs and questions to operationalize them was a multi-stage process governed not only by statistical considerations, but also by the opinions of persons with brain disorders and by clinical experts working with them. We are confident that the metric properties of the final measure with 24 questions are very good because of the thorough process with which those questions were selected and because most of them were from validated instruments and had already been tested. Our intention was to produce an original tool in terms of the development process and the scope of applicability, but without repeating the work that other authors have carried out in the past.

There have been other attempts to capture difficulties in everyday life across brain disorders. Pukrop and Moeller published a study on the development of a modular system for assessing the quality of life of persons with psychiatric disorders in 2000 [23]. More recently, Cella et al. validated 13 brief measures of quality of life, each of which comprises a set of items for persons with neurologic disorders [24]. Our metric is different from these efforts for at least three reasons. First, we developed a measure applicable for both psychiatric and neurological conditions; the development of PARADISE 24 was undertaken with persons with 9 brain disorders. Second, we developed a single metric with which cardinal comparisons can be carried out. With our metric we could assess whether the psychosocial impact of brain disorders in people’s lives changes over time and the magnitude of that change. Cardinal comparisons can also be carried out among different persons or groups of persons. In contrast, if different dimensions are assessed, it is more difficult to come up with an estimation of the magnitude of the overall change or the overall difference. Third, PARADISE 24 is a metric of the impact of brain disorders in people’s lives operationalized with PSDs. We capture the extent of actual PSDs and not a subjective evaluation of whether people are more or less satisfied with those difficulties [25]. Therefore, PARADISE 24 is not a quality of life instrument.

The 24 questions of our metric covering problems and difficulties experienced in 12 mental functions, 10 activities and participation domains, in pain and in sexual functions proved to cover the complete severity continuum of PSDs. The question that best differentiates among people who are at different levels of the continuum is the one capturing energy and drive ‘*How much of a problem did you have due to not feeling rested and refreshed during the day (e*.*g*. *feeling tired*, *not having energy)*?’ The questions that differentiate least are those addressing sexual functions ‘*How much difficulty did you have in sexual activities*?’ and independence in everyday life *‘How much difficulty did you have in staying by yourself for a few days*?’ The response thresholds of these two items are very close, indicating that they only differentiate between not having a difficulty at all and having difficulty irrespective of the magnitude. Based on this result, we could have dichotomized the response options of both questions without losing measurement precision. We decided, however, to keep them as they were because their thresholds were not disordered, and we thought that the metric would be easier to use if all questions had the same number of response options.

Practicability guided several of our decisions in the process of developing PARADISE 24. A primary goal was to develop a metric for clinical practice and for research. It can be used as an interview or be directly filled out by the persons with brain disorders, depending on the most feasible approach in the setting in which it will be used. In clinical practice, PARADISE 24 can be used as a profile of patient difficulties to guide the planning, follow-up and reporting of health-care interventions. The use of a profile of PSDs for the assignment of interventions can be especially useful in a multi-professional team [26]. Since we have demonstrated that the PARADISE-24 questions capture a single dimension and know the location of those questions in that dimension based on the analyses of this investigation, a summary score can also be created and easily transformed into an intuitive metric scale from 0 to 100 using Table 3. The summary scores will allow clinicians to estimate patients’ overall PSD levels, to monitor disease and treatment management and to follow patients along the continuum of care and over their lifespans. For researchers, PARADISE 24 with its summary score represents an ideal outcome measure for assessing the effectiveness of interventions. For policy makers, the option of creating this score based on the PSDs that are relevant to people with brain disorders and the possibility of making comparisons across disorders make PARADISE 24 a first choice instrument for cost-effectiveness evaluations. Other instruments used for that purpose, such as the Short Form-36 [27] and the EQ-5D [28] are not appropriate for people with mental disorders [29,30].

We are aware that clinicians and researchers specialized in specific brain disorders may miss PSDs they frequently see in their patients. If this is the case, we suggest adding questions addressing those PSDs to PARADISE 24 to enlarge the profile of PSDs. The use of questions from existing questionnaires reported in Cieza et al. [8] is highly recommended for this purpose. For the creation of a summary score and for comparisons, however, only the information in the 24 questions of PARADISE 24 should be considered, since only those questions have been calibrated in a single metric and are relevant across brain disorders.

Our investigation also has several limitations that should be addressed in future studies. First, our sample was a convenience sample of persons with brain disorders, and we cannot be certain about the generalisability of the results. Second, to standardize the data collection the information to answer the questions was always collected during an interview. Future studies have to determine whether the psychometric properties of the metric are still good when the persons with brain disorders fill in the questionnaire themselves. Third, we were not able to test for DIF by each of the nine conditions separately because the requirement of the procedure we used is that at least 5 observations per response option of each PSD are available for all health conditions. This was not met in our sample. To overcome this limitation we tested for DIF for psychiatric vs. neurologic disorders. Further studies with larger sample sizes should test for DIF by health conditions. Finally, we did not collected information for at least one part of the sample at two time points. Therefore, data on the sensitivity to change of the metric are still missing.

## Conclusion

A metric for the assessment of the impact of brain disorders in people’s lives has been constructed for the first time. The metric is called PARADISE 24 and is based on the hypothesis of horizontal epidemiology, which affirms that people with brain disorders commonly experience PSDs and which has been confirmed in another investigation [8]. This metric is a useful tool to carry out cardinal comparisons over time of the magnitude of the psychosocial impact of brain disorders and between persons and groups in clinical practice and research.

## Supporting Information

S1 Table PSD of the PARADISE data collection protocol and the percentage of persons reporting PSDs by health disorder (DOCX)

S1 Text PARADISE 24 –Metric of the impact of brain disorders on people’s lives, based on psychosocial difficulties that are experienced in common across brain disorders (DOCX)
