# Supplementary material for: PARADISE 24: A Measure to Assess the Impact of Brain Disorders on People’s Lives
Source: PLoS One. 2015 Jul 6;10(7):e0132410. doi: 10.1371/journal.pone.0132410 (PMC4492620; doi:10.1371/journal.pone.0132410)
Supplement: S1 Table — (DOCX) [file pone.0132410.s001.docx]

**Supplementary file 1:** Psychosocial difficulties (PSD) included in the PARADISE data collection protocol and percentage of persons reporting PSD by health disorder.

| **Psychosocial difficulties^§^** | | | **Epilepsy** | **Migraine** | **Multiple Sclerosis** | **Parkinson** | **Stroke** | **Dementia** | **Depression** | **Schizophrenia** | **Substance dependency** |
| --- | --- | --- | --- | --- | --- | --- | --- | --- | --- | --- | --- |
| **ICF Code** | **PSD Name** | **Operationalization: Question (Source*)** | **Percentage of persons reporting difficulty** | | | | | | | | |
| **Mental functions** | | | | | | | | | | | |
| b130 | Energy and drive functions | How much of a problem did you have due to not feeling rested and refreshed during the day (e.g. feeling tired, not having energy)? (WHS) | **80.0** | **95.0** | **81.3** | **75.0** | **71.3** | **83.8** | **92.6** | **86.4** | **92.5** |
| b1301 | Motivation | How much of a problem did you have not finding things that kept you interested and motivated? | **45.0** | **57.5** | **48.8** | **30.0** | **45.0** | **67.5** | **91.4** | **79.0** | **87.5** |
| b1302 | Appetite | How much of a problem did you have with your appetite? | **26.3** | **41.3** | 20.0 | 16.3 | 15.0 | **40.0** | **63.0** | **50.6** | **51.3** |
| b1304 | Impulse control | How much of a problem did you have resisting doing or saying things in ways you would normally think were inappropriate? (CIDI) | 18.8 | **52.5** | **38.8** | **30.0** | 15.0 | **37.5** | **56.8** | **60.5** | **60.0** |
| b134 | Sleep functions | How much of a problem did you have with sleeping, such as falling asleep, waking up frequently during the night or waking up too early in the morning? (WHS) | **46.3** | **71.3** | **57.5** | **70.0** | **46.3** | **57.5** | **86.4** | **72.8** | **83.8** |
| b140 | Attention functions | How much difficulty did you have in concentrating on doing something for ten minutes? (WHODAS II) | **36.3** | **62.5** | **30.0** | **38.8** | **50.0** | **41.3** | **77.8** | **56.8** | **68.8** |
| b144 | Memory functions | How much difficulty did you have in remembering to do important things? (WHODAS II) | **57.5** | **47.5** | **36.3** | **32.5** | **62.5** | **91.3** | **77.8** | **65.4** | **82.5** |
| b147 | Psychomotor functions | How much of a problem did you have with being slowed down or feeling as if things were moving too fast around you? (Based on HADS) | **33.8** | **66.3** | **50.0** | **81.3** | **65.0** | **72.5** | **80.3** | **67.9** | **65.0** |
| b147 | Agitation & Aggression / Hyperactivity | How much of a problem did you have being so irritable that you started arguments, shouted at people or even hit people? (Based on CIDI) | 20.0 | **55.0** | **41.3** | **36.3** | 25.0 | **53.8** | **75.3** | **44.4** | **71.3** |
| b152 | Emotional functions | How much have you been emotionally affected by your < health condition >? (WHODAS II) | **73.8** | **88.8** | **78.8** | **66.3** | **80.0** | **67.5** | **97.5** | **77.8** | **90.0** |
| b152 | Depressive mood | How much of a problem did you have with feeling sad, low or depressed? (WHS) | **66.3** | **81.3** | **61.3** | **63.8** | **71.3** | **76.3** | **98.8** | **81.5** | **88.8** |
| b152 | Worry and anxiety | How much of a problem did you have with worry or anxiety? (WHS) | **68.8** | **87.5** | **73.8** | **61.3** | **72.5** | **65.0** | **96.3** | **80.3** | **92.5** |
| b152 | Stress | How much of a problem did you have with not being able to cope with all the things that you had to do? (WHS) | **55.0** | **81.3** | **55.0** | **70.0** | **57.5** | **67.5** | **77.8** | **67.9** | **76.3** |
| b1521 | Regulation of emotions | How much of a problem did you have with emotional or mood swings, such as laughing or crying suddenly for no reason? (Based on HSQuale and SIP) | **35.0** | **48.8** | **37.5** | **42.5** | **52.5** | **41.3** | **66.7** | **58.0** | **87.5** |
| b156 | Perceptual functions / Sensoperceptive functions | How much of a problem did you have with hearing or seeing things that other people do not hear or see? | 6.3 | 15.0 | 8.8 | 11.3 | 3.8 | 15.0 | 12.4 | **45.7** | 23.8 |
| b152 | Feeling empty, emptiness / loneliness | How much of a problem did you have with feeling “empty” inside? (Based on CIDI) | 17.5 | **46.3** | **32.5** | **31.3** | 17.5 | **33.8** | **84.0** | **63.0** | **81.3** |
| b152 | Feeling empty, emptiness / loneliness | How much of a problem did you have with feeling lonely even when with people? (Based on SCL-90) | 22.5 | **38.8** | **40.0** | 25.0 | 22.5 | **48.8** | **79.0** | **82.7** | **77.5** |
| B1678 | Linguistic processing | How much of a problem did you have in finding the words you wanted to say or understanding words said to you? (Based on HSQuale) | **40.0** | **62.5** | **38.8** | **63.8** | **50.0** | **66.3** | **76.5** | **74.1** | **77.5** |
| b160 | Thought functions | How much of a problem did you have with feeling that your thoughts were too slow or that you could not think clearly? (SCAN) | **28.8** | **57.5** | **31.3** | **27.5** | **48.8** | **48.8** | **79.0** | **71.6** | **77.5** |
| b1602 | Irrational fears / phobias | How much of a problem did you have with feeling strong fear even though you knew there was no real danger? (Based on CIDI) | 13.8 | **35.0** | **26.3** | 18.8 | 17.5 | **27.5** | **51.9** | **61.7** | **58.8** |
| b164 | Executive functions | How much difficulty did you have in making decisions? | 25.0 | **48.8** | **36.3** | **40.0** | **28.8** | **67.5** | **74.1** | **70.4** | **78.8** |
| b164 | Executive functions | How much difficulty did you have in analysing and finding solutions to problems in day-to-day life? (WHODAS II) | 22.5 | **43.8** | 22.5 | **36.3** | **30.0** | **71.3** | **72.8** | **67.9** | **81.3** |
| **Other body functions under central neurological control** | | | | | | | | | | | |
| b2 | Sensory functions | How much of a problem did you have with sensory disturbances, such as hypersensitivity to light or noise, numbness, tingling, blurred or double vision? | **26,3** | **75,0** | **56,3** | **62,5** | **40,0** | 25,0 | **59,3** | **64,2** | **60,0** |
| b280 | Pain | How much bodily ache or pain did you have? (WHS) | **48,8** | **93,8** | **48,8** | **53,8** | **51,3** | **68,8** | **82,7** | **59,3** | **81,3** |
| b310, b320 | Speaking | How much of a problem did you have in speaking clearly? (Based on SA-SIP) | **32.5** | **45.0** | 25.0 | **47.5** | **51.3** | 15.0 | **50.6** | **46.9** | **55.0** |
| b525 | Bowel functions | How much of a problem did you have with controlling your bowels, such as being constipated or having diarrhoea? (Based on SIS/HAQUAMS) | 25.0 | **48.8** | **50.0** | **46.3** | **28.8** | **41.3** | **60.5** | **44.4** | **65.0** |
| b530 | Weight maintenance functions | How much of a problem did you have with gaining or losing weight? | 21.3 | **35.0** | **37.5** | **38.8** | 17.5 | **30.0** | **58.0** | **59.3** | **57.5** |
| b620 | Urination functions | How much of a problem did you have with passing water (urinating) or in controlling urine (incontinence)? (HSQR) | 5.0 | 11.3 | **40.0** | **38.8** | 21.3 | **36.3** | 24.7 | **33.3** | **31.3** |
| b640 | Sexual functions | How much difficulty did you have in sexual activities? (WHODAS II) | 23.8 | 23.8 | 18.8 | **28.8** | 21.3 | 7.5 | **65.4** | **27.2** | **38.8** |
|  | Libido | How much of a problem did you have with your level of sexual desire? | 16.3 | **31.3** | **32.5** | **42.5** | 17.5 | 7.5 | **81.5** | **39.5** | **51.3** |
| b730 | Muscle power functions | How much of a problem did you have with the strength of your muscles in your face, arms or legs? | 17.5 | **46.3** | **56.3** | **56.3** | **60.0** | **27.5** | **55.6** | **40.7** | **58.8** |
| b735 | Muscle tone functions | How much of a problem did you have with increased muscle tone or stiffness? | 13.8 | 22.5 | **38.8** | **71.3** | **45.0** | 18.8 | **48.2** | **48.2** | **66.3** |
| b760 | Control of voluntary movement functions, coordination, gait | How much of a problem did you have with coordinating your movements? | 17.5 | 22.5 | **47.5** | **71.3** | **60.0** | **45.0** | **45.7** | **45.7** | **61.3** |
| b7 | Balance | How much of a problem did you have with your balance? | 18.8 | **37.5** | **60.0** | **66.3** | **58.8** | **53.8** | **51.9** | **38.3** | **58.8** |
| b765 | Involuntary movement functions | How much of a problem did you have with tremor (e.g. shaking and trembling) in your arms or legs? | 21.3 | **26.3** | 22.5 | **68.8** | **31.3** | 23.8 | **45.7** | **48.2** | **61.3** |
| **Difficulties in activities and participation** | | | | | | | | | | | |
| d1 | Learning and applying knowledge | How much difficulty did you have in learning a new task, for example, learning how to get to a new place? (WHODAS II) | 15.0 | **47.5** | 21.3 | **31.3** | **31.3** | **80.0** | **50.6** | **56.8** | **65.0** |
| d166 | Reading | How much difficulty did you have in reading books, instructions or newspapers? | 16.3 | **46.3** | 25.0 | 25.0 | **31.3** | **66.3** | **70.4** | **71.6** | **56.3** |
| d230 | Carrying out daily routine | How much difficulty did you have in carrying out your day-to-day activities? | **42.5** | **68.8** | **46.3** | **45.0** | **61.3** | **72.5** | **87.7** | **66.7** | **57.5** |
| d3 | Communication | How much difficulty did you have in generally understanding what people say? (WHODAS II) | 11.3 | **36.3** | 6.3 | 16.3 | 18.8 | **61.3** | **35.8** | **64.2** | **52.5** |
| d3 | Communication | How much difficulty did you have in starting and maintaining a conversation? (WHODAS II) | 13.8 | **36.3** | 12.5 | **31.3** | **31.3** | **67.5** | **50.6** | **65.4** | **65.0** |
| d4 | Mobility (in general) | How much difficulty did you have in standing for long periods such as 30 minutes? (WHODAS II) | 12.5 | **40.0** | **42.5** | **60.0** | **48.8** | **38.8** | **63.0** | **61.7** | **62.5** |
| d430 | Lifting and carrying objects | How much difficulty did you have in lifting and carrying things? | 12.5 | **47.5** | **48.8** | **56.3** | **43.8** | **75.0** | **66.7** | **56.8** | **51.3** |
| d440 d445 | Hand and arm use and fine hand use | How much difficulty did you have in using your hands and fingers, such as picking up small objects or opening or closing containers? (HSQR) | 5.0 | 13.8 | **38.8** | **65.0** | **32.5** | **31.3** | **44.4** | **27.2** | **38.8** |
| d455 | Moving around | How much difficulty did you have with moving around? (WHS) | 5.0 | 17.5 | **27.5** | **38.8** | **40.0** | **46.3** | **37.0** | **40.7** | **40.0** |
| d450 | Walking | How much difficulty did you have in walking a long distance such as a kilometre (or equivalent)? (WHODAS II) | 17.5 | **43.8** | **41.3** | **56.3** | **51.3** | **65.0** | **48.2** | **50.6** | **46.3** |
| d475 | Driving | How much difficulty did you have in driving? | **26.3** | **27.5** | 23.8 | **37.5** | **43.8** | 13.8 | **32.1** | 13.6 | 8.8 |
| d510 | Washing oneself | How much difficulty did you have in washing your whole body? (WHODAS II) | 3.8 | 7.5 | 15.0 | 22.5 | 23.8 | **38.8** | **32.1** | **35.8** | **27.5** |
| d530 | Toileting | How much difficulty did you have in using the toilet? | 5.0 | 17.5 | 10.0 | 16.3 | 11.3 | 23.8 | 19.8 | 16.1 | 8.8 |
| d540 | Dressing | How much difficulty did you have in getting dressed? (WHODAS II) | 5.0 | 7.5 | 13.8 | **43.8** | **27.5** | **28.8** | **32.1** | 21.0 | 22.5 |
| d550 | Eating | How much difficulty did you have in eating? (WHODAS II) | 1.3 | 16.3 | 5.0 | 16.3 | 11.3 | 13.8 | **35.8** | 14.8 | 16.3 |
| d5 | Independency in everyday activities | How much difficulty did you have in staying by yourself for a few days? (WHODAS II) | 10.0 | 11.3 | 11.3 | 12.5 | **31.3** | **56.3** | **45.7** | **49.4** | **60.0** |
| d570 | Looking after one’s health | How much difficulty did you have with looking after your health, such as eating well, exercising and taking your medicines? | 5.0 | **30.0** | 23.8 | 17.5 | 18.8 | **71.3** | **48.2** | **60.5** | **66.3** |
| d640 | Doing housework | How much difficulty did you have in taking care of your household responsibilities? (WHODAS II) | 18.8 | **48.8** | **32.5** | 25.0 | **47.5** | **77.5** | **79.0** | **64.2** | **66.3** |
| d660 | Caring for others | How much difficulty did you have in providing for or supporting others? (Based on WHOQoL) | 15.0 | **42.5** | 25.0 | 23.8 | **26.3** | **57.5** | **39.5** | **51.9** | **65.0** |
| d7 | Interpersonal interactions and relationships | How much difficulty did you have in dealing with conflicts and tensions with others? (WHS) | 15.0 | **50.0** | **35.0** | **28.8** | 16.3 | **50.0** | **67.9** | **70.4** | **77.5** |
| d7 | Interpersonal interactions and relationships | How much difficulty did you have in dealing with people you do not know? (WHODAS II) | 16.3 | **37.5** | 22.5 | **32.5** | 21.3 | **57.5** | **32.1** | **69.1** | **68.8** |
| d7500 | Informal relationships with friends | How much difficulty did you have in maintaining a friendship? (WHODAS II) | 22.5 | **36.3** | 12.5 | 16.3 | 22.5 | **47.5** | **44.4** | **61.7** | **68.8** |
| d760 + d770 | Family relationships and intimate relationships | How much difficulty did you have in getting along with people who are close to you? (WHODAS II) | 10.0 | **45.0** | 20.0 | 18.8 | **28.8** | **48.8** | **35.8** | **65.4** | **58.8** |
| d839 + d850 | Education / Work and employment | How much difficulty did you have in your day-to-day work or school? (WHODAS II) | **42.5** | **55.0** | **38.8** | 22.5 | **26.3** | 25.0 | **48.2** | **35.8** | 22.5 |
| d870 | Economic self-sufficiency | How much difficulty did you have with having enough money to meet your needs? (Based on the WHOQoL) | 22.5 | **38.8** | **41.3** | **26.3** | **33.8** | **38.8** | **40.7** | **70.4** | **85.0** |
| d870 | Economic self-sufficiency | How much difficulty did you have with managing your money? | 15.0 | **30.0** | **26.3** | 17.5 | 17.5 | **50.0** | 22.2 | **64.2** | **85.0** |
| d9 | Community, social and civic life | How much difficulty did you have in joining in community activities (for example, festivities, religious or other activities) in the same way as anyone else can? (WHODAS II) | 16.3 | **51.3** | **32.5** | **28.8** | **28.8** | **51.3** | **69.1** | **60.5** | **85.0** |
| d920 | Recreation and leisure | How much difficulty did you have in doing things by yourself for relaxation or pleasure? (WHODAS II) | 16.3 | **40.0** | **26.3** | 13.8 | **28.8** | **53.8** | **75.3** | **58.0** | **80.0** |
