# Supplementary material for: PARADISE 24: A Measure to Assess the Impact of Brain Disorders on People’s Lives
Source: PLoS One. 2015 Jul 6;10(7):e0132410. doi: 10.1371/journal.pone.0132410 (PMC4492620; doi:10.1371/journal.pone.0132410)
Supplement: S1 Text — (DOCX) [file pone.0132410.s002.docx]

**Supplementary file 2:** PARADISE 24 – Metric of the impact of brain disorders on people’s lives, based on psychosocial difficulties that are experienced in common across brain disorders.

**PARADISE 24**

The following questions address problems and difficulties you might have in different functions of your body and areas of your life. Please, answer them thinking back over the past 30 days and taking both good and bad days into account.

|  | **None** | **Some** | **A lot** |
| --- | --- | --- | --- |
| 1. How much of a problem did you have due to not feeling rested and refreshed during the day (e.g. feeling tired, not having energy)? | 0 | 1 | 2 |
| 1. How much of a problem did you have with loss of interest? | 0 | 1 | 2 |
| 1. How much of a problem did you have with your appetite? | 0 | 1 | 2 |
| 1. How much of a problem did you have with sleeping, such as falling asleep, waking up frequently during the night or waking up too early in the morning? | 0 | 1 | 2 |
| 1. How much of a problem did you have being so irritable that you started arguments, shouted at people or even hit people? | 0 | 1 | 2 |
| 1. How much of a problem did you have with being slowed down or feeling as if things were moving too fast around you? | 0 | 1 | 2 |
| 1. How much of a problem did you have with feeling sad, low or depressed? | 0 | 1 | 2 |
| 1. How much of a problem did you have with worry or anxiety? | 0 | 1 | 2 |
| 1. How much of a problem did you have with not being able to cope with all the things that you had to do? | 0 | 1 | 2 |
| 1. How much bodily ache or pain did you have? | 0 | 1 | 2 |
| 1. How much difficulty did you have in concentrating on doing something for ten minutes? | 0 | 1 | 2 |
| 1. How much difficulty did you have in remembering to do important things? | 0 | 1 | 2 |
| 1. How much difficulty did you have in making decisions? | 0 | 1 | 2 |
| 1. How much difficulty did you have in starting and maintaining a conversation? | 0 | 1 | 2 |
| 1. How much difficulty did you have in walking a long distance such as a kilometre (or equivalent)? | 0 | 1 | 2 |
| 1. How much difficulty did you have in grooming or dressing, toileting or eating? | 0 | 1 | 2 |
| 1. How much difficulty did you have in sexual activities? | 0 | 1 | 1 |
| 1. How much difficulty did you have in staying by yourself for a few days? | 0 | 1 | 2 |
| 1. How much difficulty did you have with looking after your health, such as eating well, exercising and taking your medicines? | 0 | 1 | 2 |
| 1. How much difficulty did you have in initiating and maintaining a friendship? | 0 | 1 | 2 |
| 1. How much difficulty did you have in getting along with people who are close to you? | 0 | 1 | 2 |
| 1. How much difficulty did you have in your day-to-day work or school? | 0 | 1 | 2 |
| 1. How much difficulty did you have with managing your money? | 0 | 1 | 2 |
| 1. How much difficulty did you have in joining in community activities (for example, festivities, religious or other activities) in the same way as anyone else can? | 0 | 1 | 2 |
| Sum row score per column |  |  |  |
| Total row score* |  | | |

* Use Table 2 to transform the row score into a metric score between 0 and 100 with which comparisons over time and between persons or groups can be carried out.
